# Crystal structure of the *Legionella pneumophila* Lpg2936 in complex with the cofactor S‐adenosyl‐L‐methionine reveals novel insights into the mechanism of RsmE family methyltransferases

**DOI:** 10.1002/pro.3305

**Published:** 2017-10-27

**Authors:** Nikos Pinotsis, Gabriel Waksman

**Affiliations:** ^1^ Department of Biological Sciences Institute of Structural and Molecular Biology, Birkbeck London United Kingdom; ^2^ Institute of Structural and Molecular Biology, Division of Biosciences University College London London United Kingdom

**Keywords:** RsmE methyltransferase, *Legionella pneumophila*, RNA methylation, trefoil knot, S‐adenosyl‐L‐methionine

## Abstract

The methylation of U1498 located in the 16S ribosomal RNA of *Escherichia coli* is an important modification affecting ribosomal activity. RsmE methyltransferases methylate specifically this position in a mechanism that requires an S‐adenosyl‐L‐methionine (AdoMet) molecule as cofactor. Here we report the structure of Apo and AdoMet‐bound Lpg2936 from *Legionella pneumophila* at 1.5 and 2.3 Å, respectively. The protein comprises an N‐terminal PUA domain and a C‐terminal SPOUT domain. The latter is responsible for protein dimerization and cofactor binding. Comparison with similar structures suggests that Lpg2936 is an RsmE‐like enzyme that can target the equivalent of U1498 in the *L. pneumophila* ribosomal RNA, thereby potentially enhancing ribosomal activity during infection‐mediated effector production. The multiple copies of the enzyme found in both structures reveal a flexible conformation of the bound AdoMet ligand. Isothermal titration calorimetry measurements suggest an asymmetric two site binding mode. Our results therefore also provide unprecedented insights into AdoMet/RsmE interaction, furthering our understanding of the RsmE catalytic mechanism.

## Abstract 2

PDB Code(s): 5O95; 5O96

**Abbreviations used**
rRNA: ribosomal RNA
AdoMet or SAM: S‐adenosyl‐L‐methionine
MTase: Methyltransferase
PUA: PseudoUridine synthase and Archaeosine transglycosylase
SPOUT: SpoU‐TrmD
RMSD: Root Mean Square Deviation.

## Introduction

RNA methylation is an important modification of the ribosome responsible for modulating ribosomal activity. Methylation of specific bases in the ribosome subunits alters ribosomal RNA (rRNA) folding and interactions with specific proteins, resulting in global changes in levels of protein synthesis.^1^ The transfer of methyl groups to acceptor RNAs is catalyzed by RNA methyltransferases (MTases), enzymes that use S‐adenosyl‐L‐methionine (AdoMet or SAM) molecules as a source of methyl groups. The AdoMet molecule is bound in a groove at the C‐terminus of the MTase and, during the methylation process, is converted into adenosyl‐homocystein (AdoHcy). The concentration ratio AdoMet/AdoHcy in the cell is therefore important for the regulation of the enzymatic activity in MTases.^2^

All AdoMet MTases belong to the SPOUT (SpoU‐TrmD) superfamily that is conserved in bacteria and eukaryotes.^3^ They appear either as individual enzymes in prokaryotes or as enzymatic domains within larger multi‐domain proteins in eukaryotes.^4^ Several determined structures belonging to the SPOUT domain family of proteins confirmed the conserved predicted fold of a three layered α/β fold with a central β‐sheet of 5–6 strands surrounded by α‐helices on each side and a conserved C‐terminal trefoil knot.^3^ All SPOUT members found to date form dimers and even though different modes of dimerization have been found, it appears that domain dimerization is essential for substrate binding and thus enzymatic activity.^5^ In general, MTases target specific sites in the rRNA. Based on amino‐acid sequence conservation, MTases can be thus grouped into functional classes such as the ribosomal RNA small subunit methyltransferase A (RsmA) class for the methylation of the $m_{2}^{6}$A1518 and $m_{2}^{6}$A1519, the RsmB class for $m^{5}$C967, the RsmC for $m^{2}$G1207, or the RsmE class for the methylation of the $m^{3}$U1498 (numbering according to the *E. coli* 16S rRNA).^2^, ^6^ Methylation of U1498 has been shown to impact on ribosomal function and fidelity. For example, a U1498G mutation affects the formation of the first peptide bond.^6^ Also, U1498 together with other bases in ribosomal RNA helix 44 where U1498 is located have been shown to be involved in hygromycin B binding, supporting a role in the response to antibiotics.^7^

Structurally the *E. coli* RsmE MTase displays a dimeric two domain structure, a SPOUT catalytic domain and a PUA (PseudoUridine synthase and Archaeosine transglycosylase) RNA binding domain.^3^, ^8^ Likely homologues of RsmE have been reported in several pathogenic bacteria including *Legionella pneumophila*.^1^ *Legionella* bacteria utilize a type IVb secretion system (T4bSS—also known as the Dot/Icm Secretion System) to secrete several hundreds of proteins, also known as effectors, into the infected host organism.^9^ Effector proteins represent about 10% of the entire *L. pneumophila* genome comprising of about 3000 genes;^10^ therefore ribosomal activity and protein synthesis are expected to be essential for the bacterium's pathogenic mechanisms.

Here, we describe the 1.5 Å resolution structure of the MTase Lpg2639 from *L. pneumophila*. Comparison with similar structures suggests that indeed Lpg2936 is an RsmE‐like methyltransferase. To further understand the catalytic mechanism of this enzyme we determined the same structure in complex with its AdoMet ligand at 2.3 Å resolution and we characterized the interaction by isothermal titration calorimetry (ITC). While previous structures of MTases bound to AdoMet exhibited partial ordering of the ligand within its active site, the structure presented here provides a complete view of AdoMet‐binding. Also, the results of the ITC experiments reveal a unique two site binding mode with different affinities. Overall, our study provides novel insights into the catalytic mechanism in RsmE‐like methyltransferases in general and that of *Legionella* in particular.

## Results

### Overall structures

**Figure 1:**
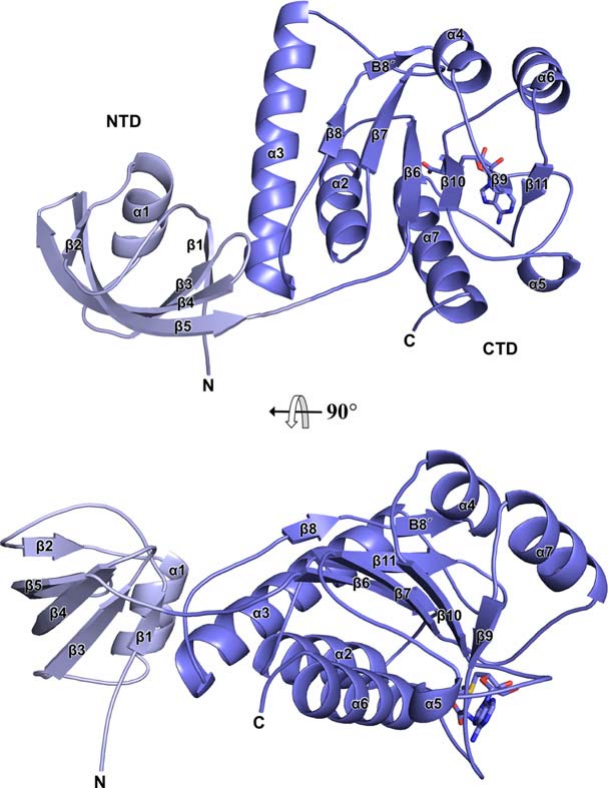
Cartoon representation of the monomeric Lpg2936. The N‐terminal and C‐terminal domains are indicated as NTD and CTD and colored in lightblue and blue, respectively. N and C‐terminal residues of the chain as well as the secondary structure elements are indicated. The bound AdoMet molecule is represented as sticks. Two orientations are shown rotated by 90°.

Two structures of the *L. pneumophila* Lpg2936 were determined: the apo (MTapo) and AdoMet‐bound structure (MTsam). The first was solved by molecular replacement using the YggJ methyltransferase from *Haemophilus influenzae* (PDB ID 1nzx) as a search model in space group *P* 2_1_. The two dimers in the asymmetric unit (AU) refined to a final *R*/*R*_free_ of 0.179/0.209 at a maximum resolution of 1.5 Å. The MTsam structure was also determined by molecular replacement using the MTapo structure as a search model in space group *I* 2 and four dimers in the AU. The MTsam structure was refined to a final *R*/*R*_free_ of 0.182/0.237 at a maximum resolution 2.3 Å (Fig. 1 and Table 1).

**Table 1:** Data collection and refinement statistics. Data for the higher resolution shell are shown in parenthesis

	MTapo	MTsam
Data Collection		
Beamline	ID30A (ESRF)	P13 (EMBL/PetraIII)
Wavelength (Å)	0.9650	0.99999
Resolution Range (Å)	47.56–1.49 (1.57–1.49)	49.4–2.30 (2.36–2.30)
Space group	*P 2_*1*_*	*I 2*
Cell parameters a, b, c, β (Å, grad)	62.78, 144.78, 64.09, 100.13	104.48, 98.74, 225.78, 91.76
Total reflections	410,121 (53,587)	392,962 (19,939)
Unique reflections	173,267 (23,778)	101,902 (5,031)
Multiplicity	2.4 (2.3)	3.9 (4.0)
Completeness (%)	95.0 (89.3)	97.0 (97.2)
Mean I/Sigma(I)	10.6 (1.1)	4.3 (1.2)
Wilson B‐factor (Å^2^)	21.6	43.0
*R*_merge_ (%)	4.4 (75.2)	17.4 (99.6)
CC1/2	0.999 (0.725)	0.983 (0.481)
Refinement		
*R*_work_/*R*_free_ (%)	17.91/20.95	18.21/23.61
CC_work_/CC_free_	0.953/0.939	0.963/0.935
Protein atoms	7,850	15,309
Solvent molecules	1,201	585
Ligand molecules	–	8 × SAM
B‐factor (Å^2^)		
Protein	23.87	42.13
Solvent	37.86	39.64
Ligand (SAM)	–	50.64
Ramachandran Plot		
Favored (%)	98.23	96.36
Allowed (%)	1.56	3.33
Outliers (%)	0.21	0.31
Clashscore	3.82	7.69
Rmsd		
Bonds (Å)	0.007	0.008
Angles (grad)	0.857	1.005
PDB code	5O95	5O96

All monomeric chains are very similar both in the MTapo and MTsam structures and they superimpose with an overall root‐mean‐square deviation (RMSD) in Cα atoms of 0.81 Å. Specifically, all eight protomers in the MTsam structure align with an average RMSD in Cα atoms of 0.51 Å while the four MTapo protomers align with the eight MTsam protomers with an average RMSD in Cα atoms of 0.60 Å, with only minor conformational changes observed near the active site (see below).

### Functional domains of the methyltransferase

Each protomer in both MTapo and MTsam structures comprises two distinct domains, an N‐terminal domain (NTD; residues 1–75) and a C‐terminal domain (CTD; residues 76–244). The NTD forms a twisted 5‐strand β‐sheet (β1‐β5) with the larger strands β4 and β5 located in the middle of the sheet. The sole α‐helix lies in the middle of a groove formed by the twisted β‐sheet (Fig. 1). This domain is highly conserved among RsmE‐like methyltransferases resembling a PUA domain, found in several proteins in bacteria, archaea, and eukaryotic proteins including *Homo sapiens*.^11^

The CTD belongs to the conserved superfamiliy of SPOUT MTases, defined by the distinctive α/β knot fold.^1^, ^3^ This domain harbours the dimerization and catalytic sites of the protein. The core of the domain comprises a single β‐sheet of six parallel β‐strands (β8–β8′, β7, β6, β10, β9, β11) surrounded by six α‐helices of various length (Fig. 1). The loop that connects the strand β11 and the C‐terminal α7 (β11/α7) forms a knot passing through the loop β9/α5 (Fig. 1).

**Figure 2:**
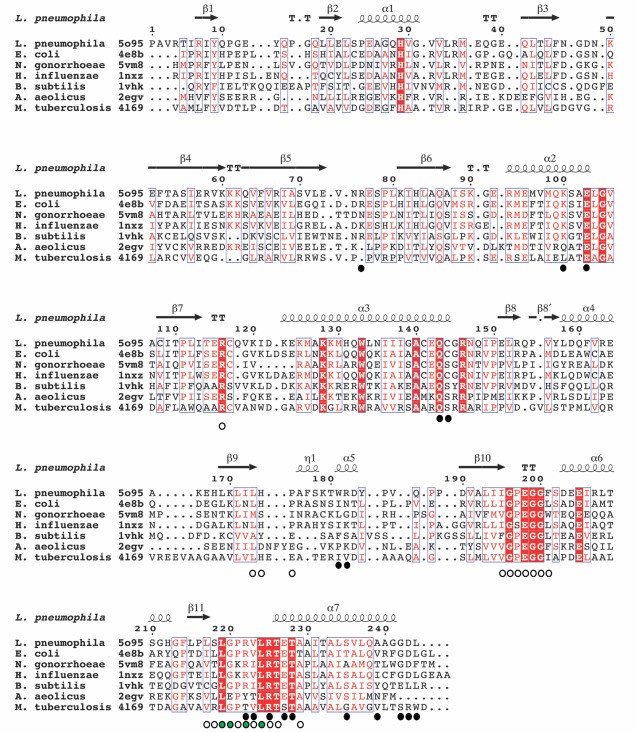
Structure‐based sequence alignment of Lpg2936 with similar RsmE methyltransferases from different bacteria. The PDB codes are indicated next to the bacteria names. Identical residues are boxed in red background and conserved residues are boxed and highlighted in red color. The secondary structure elements above the aligned sequences correspond to the *L. pneumophila* structure. The black dots indicate the dimerization interface residues and the white dots the ligand binding residues. The green dots indicate the residues involved in H‐bonding with the adenine moiety of the AdoMet molecule.

Even though NTD and CTD are clearly distinguishable, their relative orientation is invariant, forming a highly conserved assembly found in all RsmE‐like structures (see below comparison with similar structures). The interface between the NTD and the CTD covers about 520 Å^2^ of surface area in each domain and is supported by 12 H‐bonds and 4 salt bridges, involving 13 residues from the NTD and another 13 residues from the CTD (Fig. S1). The interaction in the CTD is exclusively located at the C‐terminus of the helix α3 and the loop α3/β8 (residues 131–149), while for the NTD, residues in strand β1 (particularly Arg7 and the highly conserved Tyr9), and helix α1 (Glu25 and the conserved His29) appears to play defining roles in the interface (Figs. 2 and S1).

**Figure 3:**
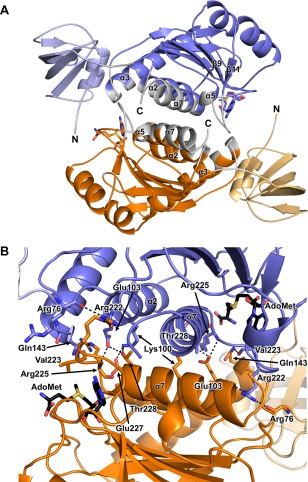
The Lpg2936 dimer. (A) Cartoon representation of the dimeric structure. One chain is colored as in Figure 1 while the second chain is colored in light orange and orange indicating the NTD and CTD, respectively. The dimerization interface is colored in white and the secondary structural elements involved are labeled. The termini of the two chains are also labeled and the AdoMet molecule is represented in sticks representation. (B) Details of residues involved in the dimerization interface. Color coding is as in panel A. The residues involved in the H‐bonding interface are labeled and colored according to the chain they belong to and the H‐bonds are represented as dashed lines. Secondary structure elements involved in the interface are also indicated. The AdoMet molecules are represented as black sticks.

Only the SPOUT domain is involved in the dimerization of the protein. The dimer interface is formed by residues in α‐helices α7, α2, loop β11/α7, and at least partially in α‐helices α3 and α5. The dimerization interface area covers an area of 1480 Å^2^ which is more than 10% of the total surface area in each monomer, suggesting a strong interaction (Fig. 3). It is mediated by 28 H‐bonds and 15 salt bridges and several of them involve interactions between main chain atoms from residues highly conserved in all SPOUT methyltransferases (Figs. 2 and 3). In detail, the interactions between O Arg76‐NH1 Arg222, NZ Lys100‐OE2 Glu227, NZ Lys100‐OG1 Thr228, OE1 Glu103‐N Arg225, OE1 Glu103‐OG1 Thr228, and NE2 Gln143‐O Val223, are symmetrically distributed at the distal ends of the interface and they are highly conserved in similar RsmE structures (Figs. 2 and 3). The core of the interface is defined by hydrophobic residues mainly located in α‐helix α7. Arg222 located in the ligand binding loop β11/α7, may play an important role, both structural and catalytic as it interacts through its side chain across the dimerization interface and through its main chain with the bound ligand (see below) (Figs. 2 and 3).

### Comparison with similar structures

A search for similar structures in the Protein Data Bank using the DALI server^12^ revealed 13 deposited entries with very similar fold belonging to 10 bacterial species. Higher scores (RSMD and Z‐score) are observed for the methyltransferase structures from *E. coli*, a fully characterized RsmE (PDB ID 4e8b),^13^ from *H. influenzae* (PDB ID: 1nxz and 1vhy)^14^ and from *N. gonorrhoeae* (PDB ID 5vm8). These structures align with the Lpg2936 structure with an RSMD in Cα atoms of 1.5–1.6 Å; however, all 13 structures display very high similarity with Lpg2936 having the same domain and dimerization architecture. Structure based sequence alignment using the most similar structures from seven different bacteria species reveals (i) a highly conserved C‐terminal part of the sequence which forms the ligand‐binding site (see below) and (ii) conserved key structural elements (including helix α3) involved in the dimerization interface and PUA‐SPOUT domain interactions (Fig. 2).

### AdoMet‐binding pocket

**Figure 4:**
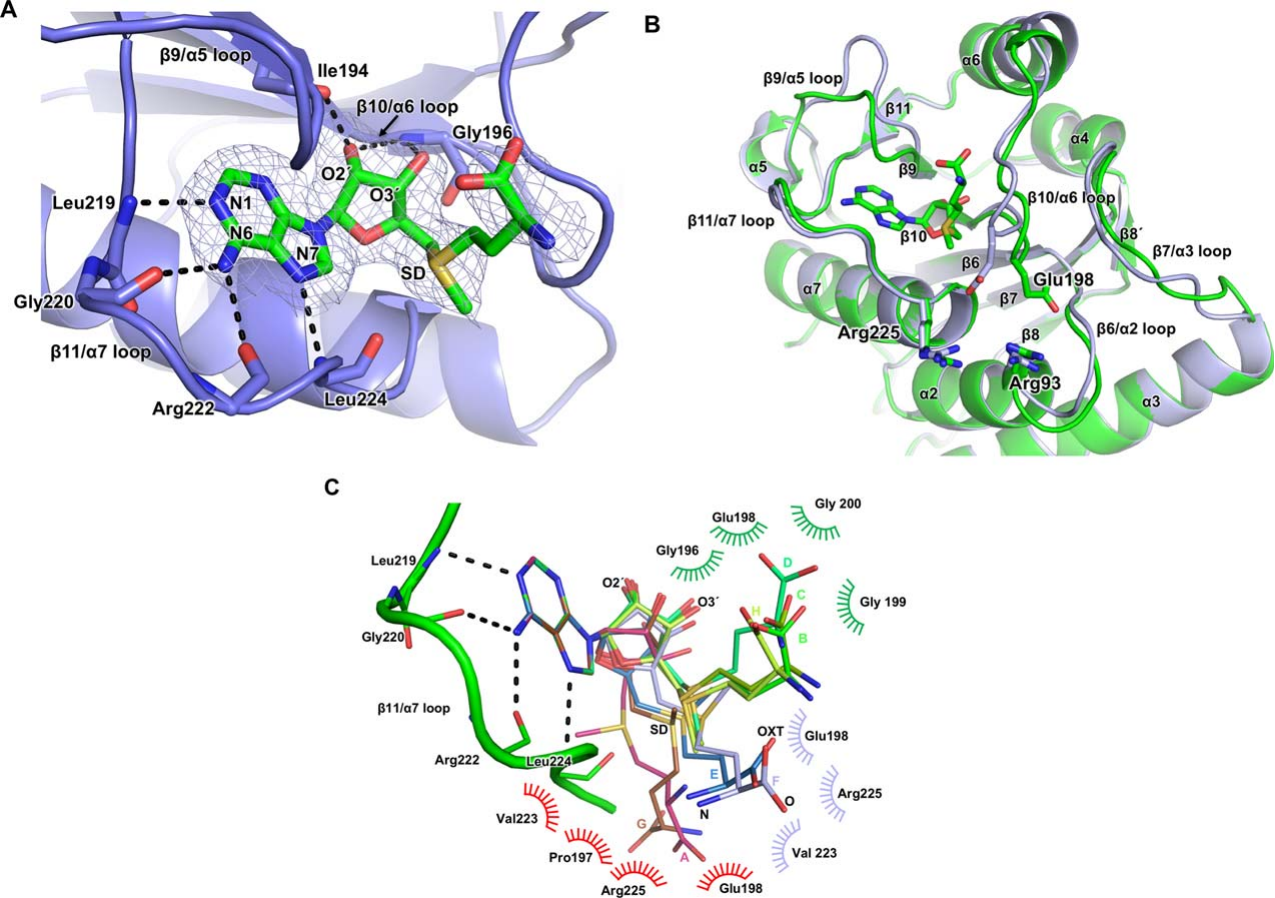
Interactions with AdoMet cofactor. (A) Cartoon and stick representation of the Lpg2936/AdoMet interactions from the chain B. A composite omit map contoured at 1.0 σ shows density for the entire AdoMet molecule (colored in green). Important loops involved in binding are shown and labeled (see main text). Residues participating in the stabilization of Adenine and Ribose moieties are shown in stick representation. H‐bonds are indicated including the residues or atoms involved. (B) Superposition of the apo (light blue) and AdoMet‐bound (green) Lpg2936 structures. The AdoMet molecule and three residues important in catalysis and mentioned in the text are shown as sticks representation. Secondary structure elements are labeled. (C) Alignment of the AdoMet molecules in all eight Lpg2036/AdeMet structures. The AdoMet molecules were aligned using the adenine moiety. Three different conformations were observed for the rest of the molecule, colored and indicated in green (chains B,C,D,H), blue (chains E,F), and red (A,G). The conserved H‐bond network stabilizing the adenine moiety is shown as black‐dashed lines. Interactions between the methionine moiety and the protein residues are grouped and colored according to the different conformations of the ligand.

A distinguishing feature of the structure presented here compared to all other structures available in the PDB is that density was observed for the entire ligand and thus a complete model of AdoMet bound to a MTase could be built for the first time [Fig. 4(A)]. The co‐factor binding pocket of the Lpg2936 lies at the C‐terminal part of the SPOUT domain. Interactions with the ligand involve residues in three conserved loops: two highly conserved ones, β10/α6 and β11/α7, and another only moderately conserved, β9/α5 [Figs. 2 and 4(A)]. Binding of the AdoMet ligand to the enzyme promotes conformational changes in the loops β9/α5 and β10/α6, but none in the β11/α7 loop, likely due to its involvement in the dimer interface [Figs. 3(B) and 4(B)]. Minor conformational changes are observed in the neighboring loops β6/α2 and β7/α3, possibly as a result of the β10/α6 loop movement [Fig. 4(B)]. To ascertain whether the observed conformational changes are due to ligand binding, and not crystal packing, we examined all protein interfaces using the PDBePISA server.^15^ Contacts with neighboring molecules in the proximity of the active site were found only in the MTsam structure and involved residues in the β9/α5 loop only in chain B. Since no such contact is observed in any other chain, we conclude that crystal packing does not affect ligand binding.

There are important conformational variations in AdoMet‐binding among the eight molecules of MTase‐AdoMet complexes in the asymmetric unit [Fig. 4(C)]. Indeed, each AdoMet molecule can be divided into two parts, one comprising the adenine group which remains conformationally invariant and the other comprising the rest of the molecule which adopts widely different conformations [Fig. 4(C)]. The AdoMet invariant part is stabilized by four H‐bonds between the adenine group and main chain atoms in the loop β11/α7 [Table 2 and Fig. 4(B)]. The variant part of the molecule encompasses the ribose group which adopts two conformations, one observed for the chains B, C, D, F, and H and one observed for the chains A, E, and G. In all chains except chain E there is at least one H‐bond between the hydroxyl groups of the ribose and the main chain atoms of Leu173 and Gly196 in the β10/α6 loop [Table 2 and Fig. 4(C)]. The methionine moiety is the most flexible part of the AdoMet molecule and can be sorted into three different conformations stabilized by three different groups of residues [indicated in Fig. 4(C) in green, blue, and red, respectively), the most frequently encountered observed in chains B, C, D, and H, while the two others are observed in chains E and F and in chains A and G, respectively [Fig. 4(C)]. For the first group, there is a positional deviation of 0.4–0.6 Å for the methyl‐thioether group that results in a 2.0–2.5 Å deviation for the carboxylic‐acid part of the methionine. This orientation exposes the methyl of the methyl‐thioether group towards a region formed by residues Arg93, Glu198, and Arg225 which have been suggested to be key residues for the interaction with U1498.^5^ For the AdoMet's methionine moieties interacting with chains E,F and A,G the deviations in the ribose group are ranging from 0.5 to 1.0 Å and 1.0 to 1.5 Å, respectively, which results in conformational changes up to 8 Å for the carboxylic part of the methionine group. The three different conformations of the AdoMet molecules (B,C,D,H—E,F—A,G) are further stabilized by hydrophobic interactions involving the AdoMet methionine moiety, which makes interactions with protein residues that are partially conserved in the three orientations.

**Table 2:** *Summary of ligand‐protein interactions. H‐bonds are defined based on the criteria in the PISA server (distance between donor and acceptor <3.9 Å)*.^15^ *Error in coordinates from the Luzzati plot is 0.3 Å*

Chain	A	B	C	D	E	F	G	H
N1‐N L219	3.2	2.9	3.0	3.2	3.2	3.1	2.8	2.8
N6‐O Gly220	2.9	2.8	3.0	2.8	3.0	3.0	2.8	3.1
N6‐O Arg222	3.3	2.8	3.1	3.0	2.4	2.8	2.9	2.9
N7‐N Leu224	3.5	3.0	3.1	3.0	2.9	3.1	3.1	3.1
O2'‐O Leu173	–	2.4	2.4	3.0	3.8	2.3	3.4	2.7
O2'‐N Gly196	–	3.2	3.7	3.4	–	–	–	3.3
O3'‐N Gly196	3.3^a^	3.3	3.3	3.7	3.5	–	3.3	3.5
SD‐O Leu224	3.8	3.4	3.3	3.5	–	3.4	3.6	3.4

a (N Gly200).

### Thermodynamic characterization of AdoMet binding

**Figure 5:**
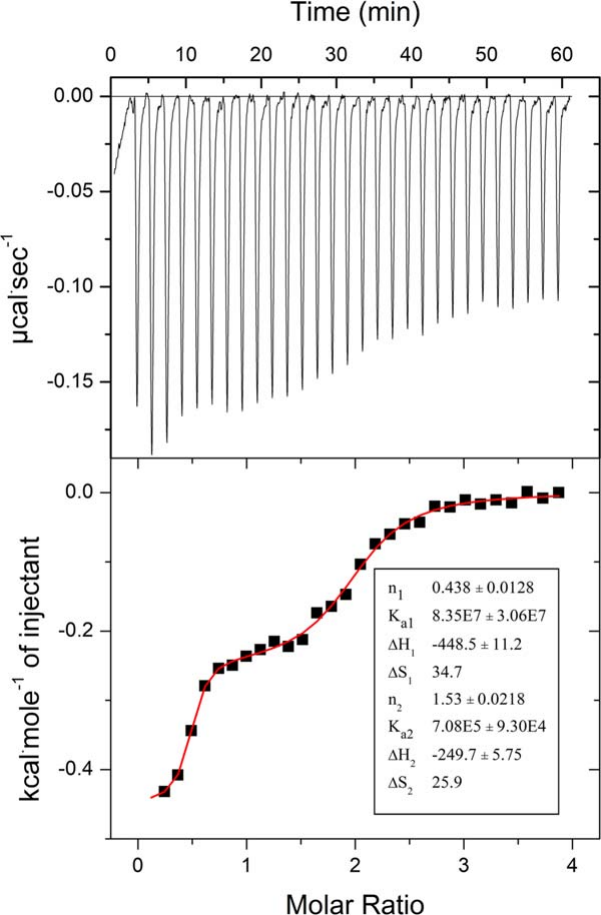
Isothermal titration calorimetric AdoMet binding to Lpg2936. The raw data are presented on the top of the panel, while the bottom panel displays the fit to the data. The square dots indicate the experimental data (including subtracted data from a blank measurement). The inset panel displays the binding thermodynamic parameters obtained using the Origin software with the *K*_a1_ value representing the first association constant and *K*_a2_ the second association constant.

To further understand the binding of the AdoMet substrate to the Lpg2936 protein we used isothermal titration calorimetry (ITC) to measure the binding thermodynamics (Fig. 5). Even though the released heat upon AdoMet binding to the protein was relatively small, a clear two binding site model was best fit to the experimental data, with a high first association constant of *K*_a1_ = 8.35 ± 3.06 × 10^7^ M^−1^ and a lower second one *K*_a2_ = 7.08 ± 0.93 × 10^5^ M^−1^. The enthalpy (ΔH) values for the two binding sites were −448.5 ± 11.2 and −249.7 ± 5.75 cal·mol^−1^ and the entropies 34.7 and 25.9 cal·deg^−1^·mol^−1^, respectively (Fig. 5). The first association constant is two orders of magnitude higher compared to the values observed for other methyltransferases such as the RsmC^16^ and RlmI.^17^ However, both these enzymes display a single binding site model for AdoMet with *K*_a_ values of 2.09 × 10^5^ and 3.4 × 10^5^ M^−1^, respectively, which are very similar to the value for *K*_a2_ observed in Lpg2936. *E coli* RsmE, like Lpg2936, exhibits a sequential binding mode with similar *K*_a1_ values but a significantly lower second association constant.^13^ Stoichiometries estimated from ITC data for the Lpg2936/AdoMet interaction is about 0.4 and 1.6 for the 1st and 2nd binding events, respectively. This is unorthodox although a model invoking a first high‐affinity binding event followed by a second low‐affinity binding event impacting on the first one might explain such observation. This remains to be explored.

## Discussion

In the current study, we report the structure of the Lpg2936 from *L. pneumophila* in presence and absence of its enzymatic substrate AdoMet. The ligand free structure was determined at high resolution. The dimeric enzyme is similar to several structures of the RsmE‐like fold including the archetypal RsmE methyltransferase from *E. coli* and the Rv2372 methyltransferase from *M. tuberculosis*.^5^, ^13^ *E. coli* RsmE is known to methylate the $m^{3}$U1498 position in the 16S ribosome RNA. Since Lpg2936 and U1498 are highly conserved across bacteria (Figs. 2 and S2), it is very likely that Lpg2936 can also function as an RsmE methyltransferase targeting the same base in the 16S rRNA of *L. pneumophila*.

RNA methyltransferases react either with unstructured RNA or ordered RNA in fully assembled ribosomal structures. The RsmE enzymes belong to the group of methyltransferases that act almost exclusively in assembled 30S ribosome subunits.^18^ A peak of activity in RsmE enzymes is observed in the presence of NH_4_Cl pH 7–9 and Mg^2+^ which were shown to stabilize the 30S subunit.^19^ It is therefore apparent that RsmE is dependent on the presence of the majority of small subunit ribosomal proteins to structure the RNA.^18^ On the other hand, the RsmE enzyme from *M. tuberculosis* also methylates the *E. coli* ribosomal RNA emphasizing the importance of sequence and structural conservation both in the enzyme and RNA sequences (Figs. 2 and S2), but also in the overall tertiary RNA structure in the assembled ribosomes.^5^, ^18^ To better understand the RsmE/RNA binding mode, we generated an electrostatic surface representation for the Lpg2936/AdoMet dimer. As is observed in other similar RsmE structures one face of the dimeric enzyme is positively charged [Fig. S3(A)] while the opposite side is negatively charged [Fig. S3(C)]. Previous molecular modeling of an RsmE enzyme with 16S RNA proposes that the observed charge distribution is essential in order to direct helix 44 where U1498 is present towards the catalytic center of the RsmE enzyme [Fig. S3(B)].^5^ In this model, Kumar et al. suggest that three conserved residues (equivalent in Lpg2936 to Arg93, Arg225 and Glu198) are involved in RNA binding and possibly in catalysis. Remarkably all Arg93 and Arg225 side chains in both structures (in total 12 chains) display the same conformation while the side chain of Glu198 adopts different conformations depending on the position of the AdoMet molecule [Fig. 4(B)] (see next paragraph).

Previous attempts to crystallize an RsmE/AdoMet complex either failed or resulted to a truncated ligand where its carboxylate moiety was missing as in the case of the *A. aeolicus* structure (PDB ID 2egv), consistent with reports that AdoMet molecules are unstable for *in vitro* experiments.^20^, ^21^ For the Lpg2936, however, all eight copies of the enzyme in the asymmetric unit are fully occupied with the AdoMet molecule. In all monomers, the adenine group of the ligand forms a very conserved interaction pattern with the enzyme and specifically with residues in the loops β11/α7 and β10/α6 [Fig. 4(C) and Table 2]. On the other hand, the carboxylate moiety of the ligand is found in three different conformations randomly found within the dimers [Fig. 4(C)]. Interestingly, in the chains B, C, D, and H the methyl‐group of the methyl‐thioether group is facing towards the residues Arg93, Arg225, and Glu198, thus indicating that in these chains, AdoMet is observed in the most favorable conformation to execute catalysis.^5^ The importance of the Arg225 is also highlighted in the *E. coli* RsmE (Arg223) where an alanine mutation renders the enzyme inactive.^13^

The ITC results presented here suggest that the interaction between Lpg2936 and AdoMet is best described as a two binding site model indicating two non‐identical binding sites. For the case of *E. coli* RsmE, a sequential binding mode was proposed,^13^ also likely applicable to Lpg2936. These binding modes are also consistent with the recent RsmE/AdoMet structure from *N. gonorrhoea* where only one of the two ligand binding sites in the dimeric structure was occupied (PDB ID 5vm8). Nevertheless, even though this asymmetric pattern of binding is observed for the Lpg2936, there are also significant differences with the other two enzymes (*E. coli, N. gonorrhoea*). In the case of the *Legionella* methyltransferase, the first association constant is significantly higher than any one observed before in any methyltransferase containing a SPOUT domain. The second association constant in *Legionella* is at similar levels to values measured for one binding (symmetrical) site in other methyltransferases. These values are in agreement with the fact that all eight binding sites in the structure are occupied implying a strong binding of AdoMet molecules. However, our structure does not exhibit obvious conformational differences between binding sites and therefore the structural basis for two sites with widely different affinities remains unclear.

The high association constants observed in the Lpg2936 may also suggest a specific role of this enzyme in the *Legionella* bacterium. A previous report indicated a 5–10% translocation efficiency for Lpg2936, suggesting that Lpg2936 might be a potential protein effector.^10^ The determined crystal structure however clearly suggests a RsmE fold and since such enzymes require a very specific substrate only present in bacterial 16S RNAs, it is highly unlikely that Lpg2936 could target eukaryotic ribosomes. It is therefore more plausible to envisage a role for Lpg2936 within the *Legionella* bacterium, through specific methylation of the *Legionella* 16S RNA subunit during infection when large amount of protein effectors need to be produced.

## Materials and Methods

### Cloning, expression, and purification of Lpg2936

The *Lpg*2936 DNA (AAU28982) encoding the wild type protein was cloned in a pETM14 vector (EMBL) using a PCR‐based in‐fusion HD cloning system (Clontech Laboratories). The expression cassette contained an N‐terminal hexa‐histidine tag followed by a 3C protease cleavage site.

The recombinant protein was over‐expressed in the bacterial strain BL21(DE3) using a previously described auto‐induction protocol.^22^ The cells were harvested by centrifugation (6000 g, 15 min) and resuspended in a lysis buffer [25 m*M* Tris‐HCl pH 7.5, 0.3 *M* NaCl, 5 m*M* β‐mercaptoethanol (βME), 10 m*M* imidazole, 5% glycerol, a tablet of protease inhibitors (Complete, EDTA‐free by Roche)] to which 0.25 mg·mL^−1^ lysozyme was added. Cells were lysed in an EmulsiFlex‐C3 homogeniser (Avestin) and the crude extract was centrifuged at 50,000 g for 45 min. The supernatant was loaded onto a 5 mL HisTrap column (GE Healthcare) equilibrated in the lysis buffer. Washing steps were performed with extended volumes of lysis buffer though the column as well high salt buffer (25 m*M* Tris‐HCl pH 7.5, 1 *M* NaCl, 5 m*M* βME, 10 m*M* imidazole, 5% glycerol). After washing, a 5 mL lysis buffer was loaded in the column containing in addition 0.04 mg·mL^−1^ 3C protease and left for 8 h to cleave the Histidine tag of the overexpressed protein. The cleaved protein was eluted with 10 mL lysis buffer, while any un‐cleaved protein was eluted with a 0.6 *M* imidazole containing lysis buffer. The protein was then concentrated to a 3 mL volume and loaded onto a Superdex 200 16/60 column (GE healthcare) equilibrated with a SEC‐buffer (25 m*M* Tris‐HCl pH 7.5, 0.15 *M* NaCl, 5 m*M* βME, 5% glycerol). A GSTrap FF 1 mL column was connected in line prior to the Superdex column to remove the 3C protease from the sample. The protein was eluted in a single peak with an apparent molecular weight of approximately 54 kDa, matching the molecular weight of a dimer. Protein quality was assessed by SDS‐PAGE suggesting a single band with protein purity better than 99%. The protein was further concentrated to 20 mg·mL^−1^ in a SEC‐buffer for crystallization screening.

#### Protein crystallization

Initial crystallization screens were performed using the sitting‐drop vapor‐diffusion technique at 16°C by mixing 0.2 μL of protein and precipitant at ratios 1:1 and 1:2. For the Lpg2936/AdoMet crystallization, prior to setting up the screenings, 0.37 m*M* (20 mg·mL^−1^) of the protein were mixed at a molar ratio 1:5 with AdoMet (50 m*M* stock solution in SEC‐buffer, Sigma Aldrich, cat # A7007). The protein was incubated with AdoMet for at least 6 h on ice and then used directly in crystallization screens. For the unbound structure crystals appeared after 3–5 days in several different conditions and for the optically most promising of them the crystals were further optimized using a hanging drop vapor diffusion setup. The best crystals were observed in a precipitant optimized from the D2 condition of the Morpheus screen^23^ containing 0.02 *M* of each alcohol, 9% w/v PEG 8,000, 18% v/v Ethylene glycol and 0.1 *M* MES/Imidazole buffer pH 6.5. The protein crystals with the bound AdoMet were directly obtained from the C1 condition of the Morpheus screen containing 10% w/v PEG 20,000, 20% v/v PEG MME 550, 0.03 *M* of each sodium nitrate, disodium hydrogen phosphate and ammonium sulfate (NPS mixture), and 0.1 *M* MES/imidazole buffer pH 6.5. The harvested crystals were directly cryo‐cooled in liquid nitrogen.

### Data collection and refinement

All data sets were collected at 100 K. Crystals of the ligand‐free protein were automatically measured at the ESRF automated beamline ID30A‐1/MASSIF‐1 (Grenoble, France) and diffracted to a maximum resolution of 1.49 Å. The protein/AdoMet crystals were measured at the PetraIII P13 beam‐line (EMBL‐Hamburg/DESY, Germany)^24^ and diffracted to a maximum resolution of 2.30 Å. All data sets were indexed, processed and scaled using the XDS package^25^ (Table 1).

The ligand‐free methyltransferase crystals belonged to the *P* 2_1_ space group with a solvent content of 52.9% corresponding to four molecules per asymmetric unit (AU). The structure was determined by molecular replacement using the HI0303 methyltransferase from *Haemophilus influenzae* as a search model (PDB ID 1nzx).^14^ After several iterations of rigid‐body, maximum‐likelihood and TLS refinement using the PHENIX suite,^26^ manual building and model inspection using COOT,^27^ a model was obtained converging to a final *R*_work_/*R*_free_ of 0.1791/0.2095. All four chains in the model cover the protein sequence starting from residues three or four up to the very last C‐terminal residue 244. The model contains in addition 1201 water molecules.

The AdoMet bound crystals belonged to the *I* 2 space group with a solvent content of 53.6% corresponding to eight molecules per AU. All eight chains were determined by molecular replacement using the unbound structure as a search model. Extra difference density at the C‐terminus of each chain was interpreted as an AdoMet molecule and it was built in COOT. After several iterations of refinement and manual building using the same strategy as for the AdoMet free protein, a model was obtained converging to a final *R*_work_/*R*_free_ of 0.1821/0.2361. All chains start from residues two or three and the amino acid sequence is visible to all up to the final residue 244. The model contains in addition 585 water molecules.

Refinement statistics for both structures are summarized in Table 1.

### Data analysis and bioinformatics

Analysis of the protein‐protein interfaces was performed by the on‐line server PDBePISA(EMBL‐EBI).^15^ Structural alignment (Cα alignment) of the Lpg2936 models with known PDB entries and sequence alignment and assignment of secondary structural elements were performed by PDBeFOLD.^28^ Representation of sequence alignments (shown in Fig. 2) using the output of PDBeFOLD was performed by the ESPript server.^29^ Molecular visualization of the crystal structures was done by PyMOL (https://pymolwiki.org/).

### Isothermal titration calorimetry

ITC was applied to quantitatively determine the binding affinity of Lpg2936 to AdoMet. For the titration experiments, the purified protein was dialyzed overnight against a buffer containing 50 m*M* HEPES pH7.5, 0.15 *M* NaCl, 5% (v/v) glycerol, 2 m*M* MgCl_2_, and 2 m*M* β‐mercaptoethanol. The AdoMet ligand was dissolved in the same buffer as the protein. The ITC experiments were carried out using a MicroCal VP‐ITC calorimeter (Malvern) at 22°C using 500 μ*M* AdoMet in the injector and 36.5 μ*M* Lpg2936 (quantified in the dimeric form) or buffer in the sample cell. Before measurements all samples were thoroughly degassed. Injection volumes of 10 μL per injection were used for each measurement, and the consecutive injections were separated by 5 min to allow the peak to return to the baseline. The heat of dilution was estimated by the last four injections after subtracting the blank titrations. The final integrated data were fitted using a two binding site model as implemented in the MicroCal Origin 7.0 software package (OriginLab Corp.).

## Supporting information

Supporting Information
