# Supplementary material for: Crystal structure of the Legionella pneumophila Lpg2936 in complex with the cofactor S‐adenosyl‐L‐methionine reveals novel insights into the mechanism of RsmE family methyltransferases
Source: Protein Sci. 2017 Oct 27;26(12):2381–91. doi: 10.1002/pro.3305 (PMC5699498; doi:10.1002/pro.3305)
Supplement: Supplementary file 1 — Supporting Information [file PRO-26-2381-s001.pdf]

SUPPLEMENTARY MATERIAL

Crystal structure of the *Legionella pneumophila* Lpg2936 in complex with the cofactor S-adenosyl-L-methionine reveals novel insights into the mechanism of RsmE family methyltransferases

Nikos Pinotsis[1] and Gabriel Waksman[1,2*]

[1]Institute of Structural and Molecular Biology, Department of Biological Sciences, Birkbeck, Malet Street, WC1E 7HX London, United Kingdom and [2]Institute of Structural and Molecular Biology, University College London, Gower Street, WC1E 6BT London, United Kingdom.

* Address for correspondence: g.waksman@ucl.ac.uk and g.waksman@mail.cryst.bbk.ac.uk

Running title: Crystal structure of the *Legionella* effector Lpg2936

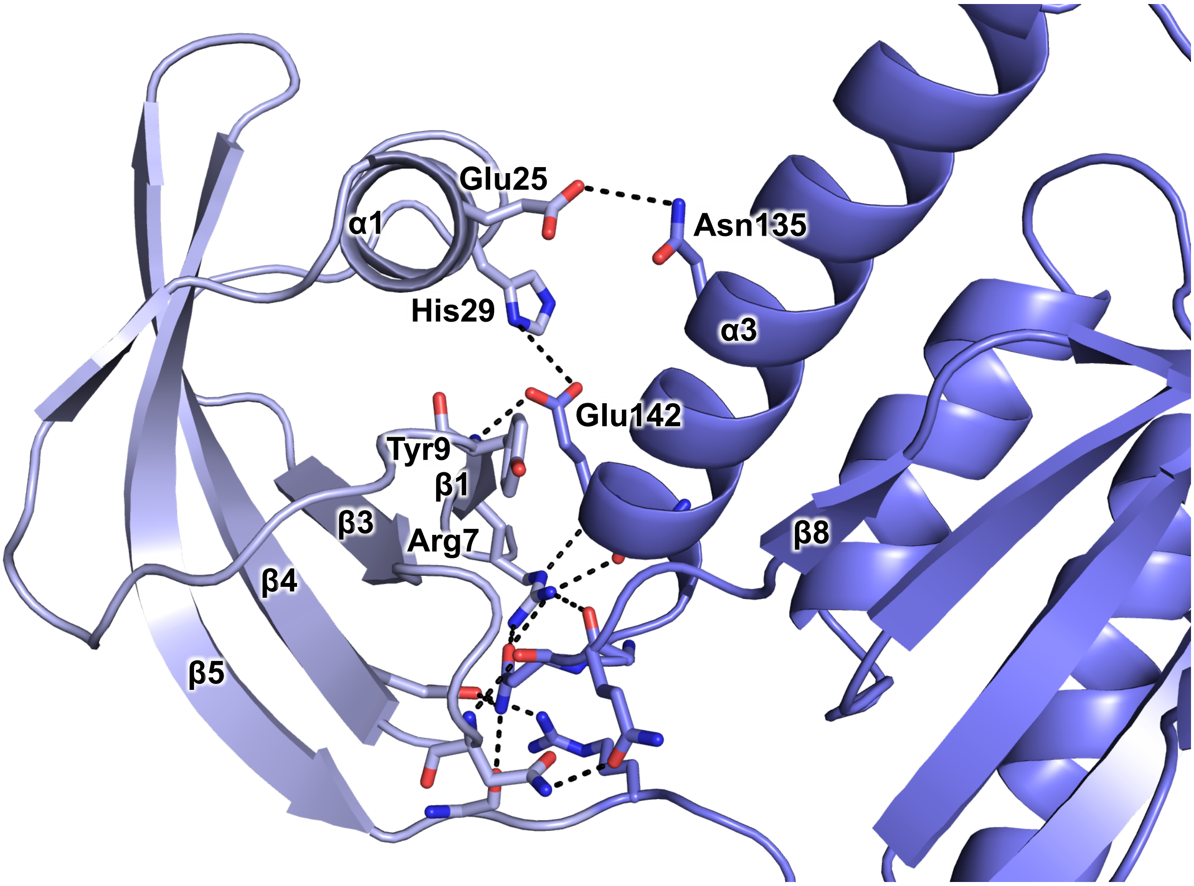

Figure S1: Cartoon and stick representation of the interactions between the N-terminal and C-terminal domains. Color codes are the same as in Figure 1. H-bonds are represented as black dashed lines. Residues involved in the interactions and secondary structural elements are labeled.

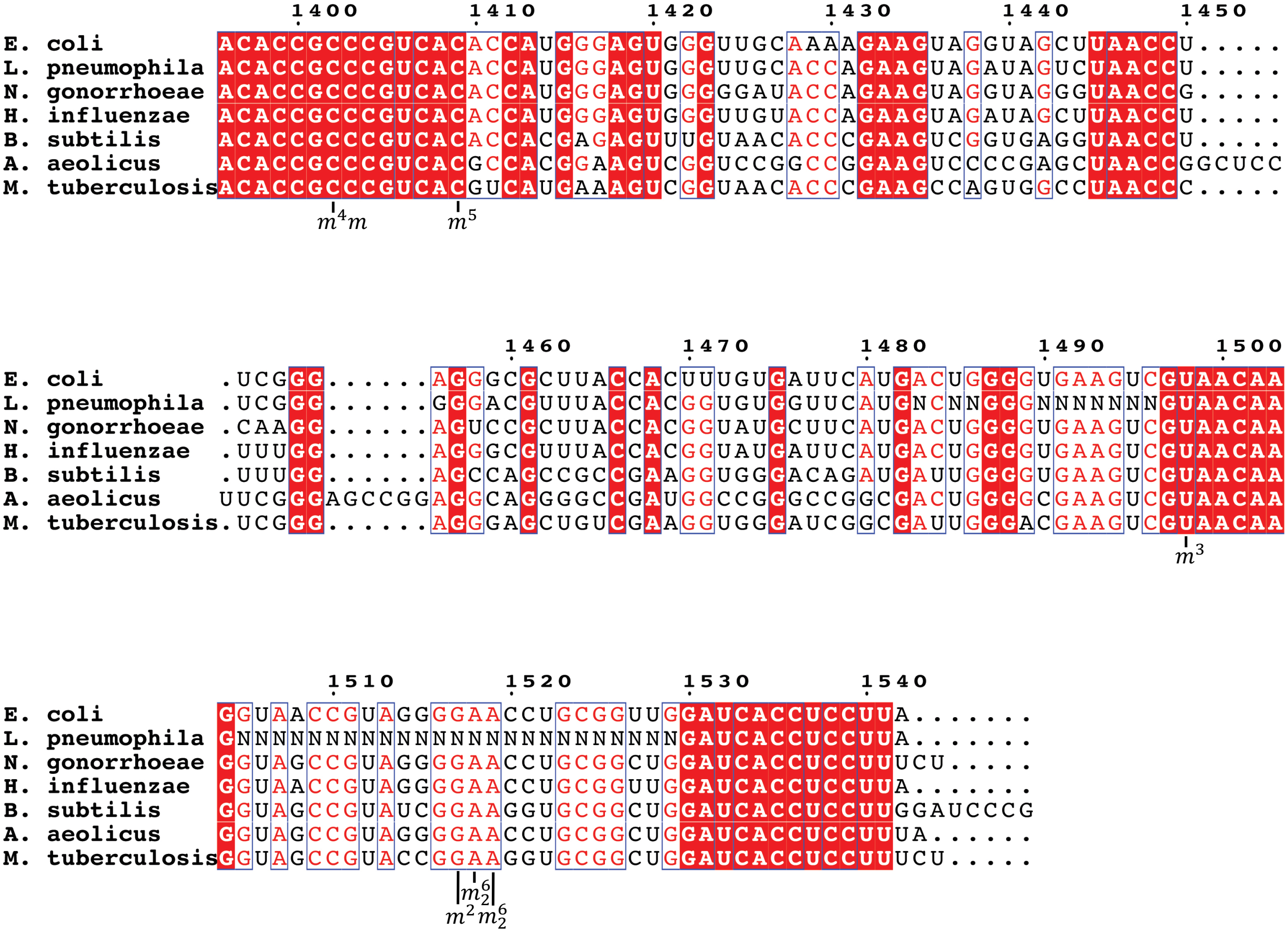

Figure S2: Sequence alignment of the 16S RNAs from different organisms. The methylation positions for different methyltransferases are indicated. The numbering is according to the *E. coli* 16S rRNA.

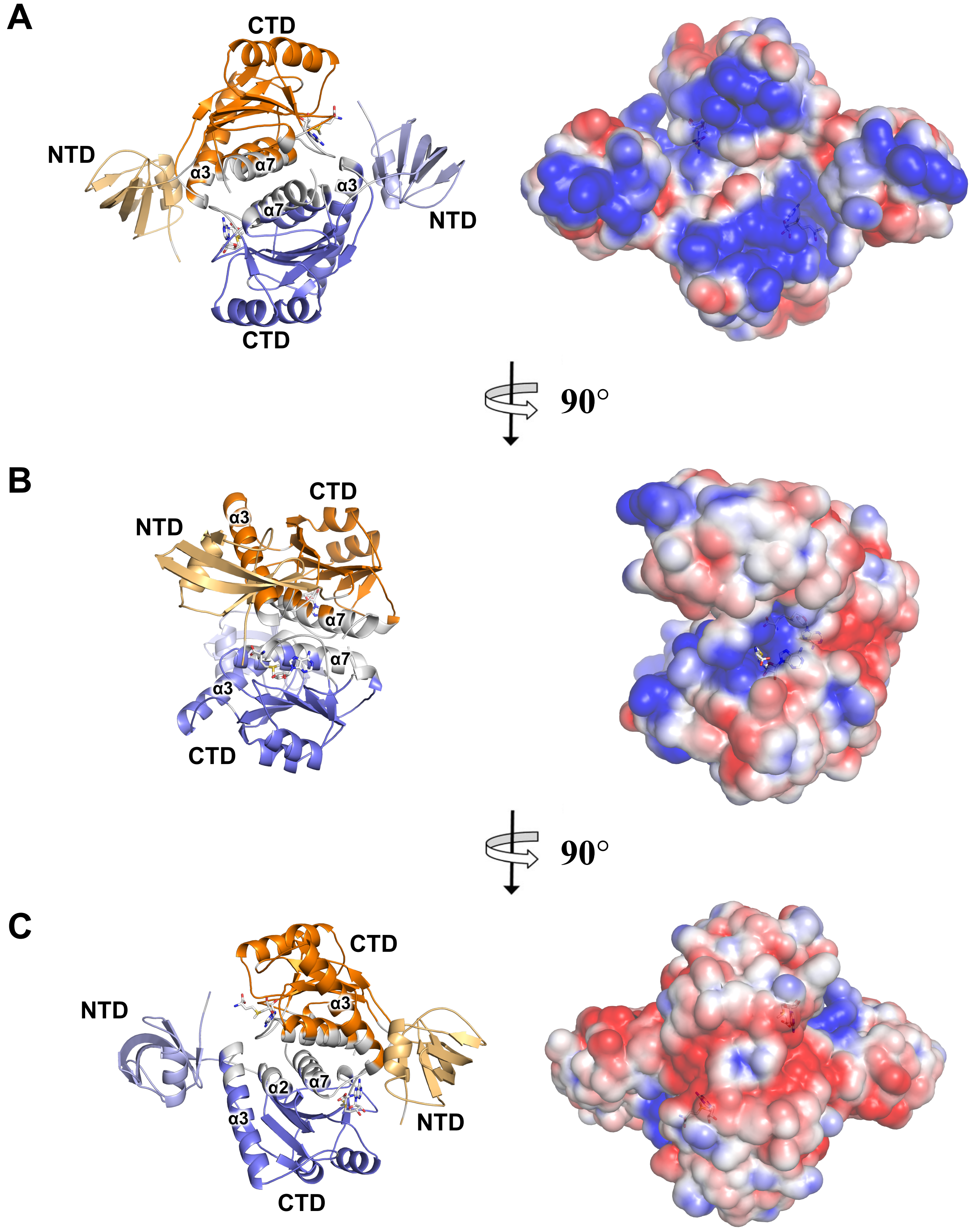

Figure S3: Electrostatic representation of Lpg2936 in three different orientations rotated by 90 degrees. The N- and C-terminal domains are indicated as NTD and CTD, respectively. Colour coding and labelling of panels at left is as in Fig. 3A. The surface gradient is coloured with a blue to red gradient from $-1.5k_BT/e$ to $+1.5k_BT/e$.
